# Wettability Gradient-Induced Diode: MXene-Engineered Membrane for Passive-Evaporative Cooling

**DOI:** 10.1007/s40820-024-01359-8

**Published:** 2024-03-21

**Authors:** Leqi Lei, Shuo Meng, Yifan Si, Shuo Shi, Hanbai Wu, Jieqiong Yang, Jinlian Hu

**Affiliations:** Department of Biomedical Engineering, City University of Hong Kong, Hong Kong S. A. R, 999077 China

**Keywords:** Passive-evaporative cooling, MXene, Electrospun membrane, Wettability gradient, Diode

## Abstract

Thermoregulatory textiles, leveraging high-emissivity structural materials, have arisen as a promising candidate for personal cooling management; however, their advancement has been hindered by the underperformed water moisture transportation capacity, which impacts on their thermophysiological comfort. Herein, we designed a wettability-gradient-induced-diode (WGID) membrane achieving by MXene-engineered electrospun technology, which could facilitate heat dissipation and moisture-wicking transportation. As a result, the obtained WGID membrane could obtain a cooling temperature of 1.5 °C in the “dry” state, and 7.1 °C in the “wet” state, which was ascribed to its high emissivity of 96.40% in the MIR range, superior thermal conductivity of 0.3349 W m^−1^ K^−1^ (based on radiation- and conduction-controlled mechanisms), and unidirectional moisture transportation property. The proposed design offers an approach for meticulously engineering electrospun membranes with enhanced heat dissipation and moisture transportation, thereby paving the way for developing more efficient and comfortable thermoregulatory textiles in a high-humidity microenvironment.

**Supplementary Information:**

The online version contains supplementary material available at 10.1007/s40820-024-01359-8.

## Highlights

- Engineering MXene into electrospun nanofibers can effectively enhance its thermal emissivity and conductance, and the unidirectional water transport of the wettability-gradient-induced-diode (WGID) membrane displayed diode-like properties with wettability gradient by tailoring the water contact angle of each single layer.
- The WGID membrane could achieve a cooling temperature of 1.5 °C in the “dry” state, and 7.1 °C in the “wet” state, with high emissivity of 96.40% in the MIR range, superior thermal conductivity of 0.3349 W m^−1^ K^−1^.
- Zero-energy-consumption for personal cooling management via multiple heat dissipation pathways, including thermal radiation, conduction, and evaporation.

**Supplementary Information:**

The online version contains supplementary material available at 10.1007/s40820-024-01359-8.

## Graphical abstract

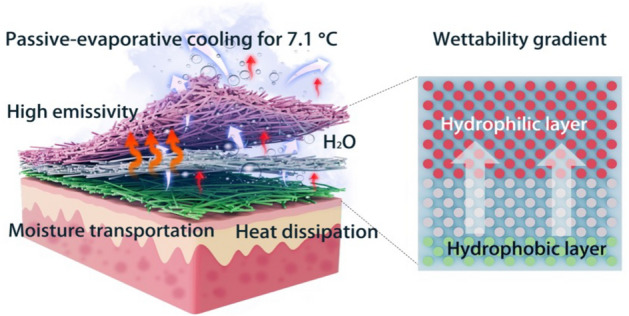

## Introduction

Maintaining thermal comfort for human body is crucial for basic metabolism and efficient thermal management. Modern buildings typically make use of electronic systems to ensure individual thermophysiological comfort, which needs huge energy to cool the entire space [1–5]. Basically, the heat dissipation routes of the naked human body primarily focus on conduction-, radiation- and evaporation-controlled mechanisms based on the thermoregulatory textiles, of which radiative-evaporative heat dissipation accounts for more than 70% portions [6–8]. The existing design of temperature-regulated textiles relies on a single coefficient of heat loss, leading to the inadequate thermophysiological comfort for the human body. Recently, there has been a great deal of interest in the creation of thermoregulatory clothing that provide different degrees of passive cooling effect (PCE) via a radiation-controlled mechanism [9–11]. However, such textiles with moisture-wicking behavior fail to be unidirectional, and allow water moisture to penetrate in the reverse direction, due to their limited water-transportation capacity and relatively slow evaporation rate of water moisture. Consequently, the aforementioned drawbacks may trigger sticky and chilly sensations on the human skin, especially with prolonged exposure leading to perspiration deposition, which significantly impacts on the temperature-regulated effects and thermal comfort of the human skin [3, 12–14]. Thus, passive-evaporative cooling clothing remains a vital component of PCE in order to satisfy the thermophysiological comfort for the human body, which is attributable to effective conveyance of perspiration-driven moisture and temperature-regulated skin microenvironment [15–17].

Previous studies have proven a range of fiber-based textiles, effectively eliminating perspiration or moisture from the skin and mitigating the sensation of wetness [5, 18–21]. Nevertheless, these thermoregulatory textiles consist of either hydrophilic layers, which keep the skin moist for long periods of time, or hydrophobic layers, which are incapable of transporting perspiration-driven water from human skin [22, 23]. Furthermore, considerable efforts have been devoted to the design and development of unidirectional water moisture-wicking materials based on the Janus wettability, a typical protocol with a direct shift in wettability is produced by integrating two layers with asymmetric wettability [6, 24–26]. However, the progressed Janus membranes might encounter a reverse penetration during the process of consecutively conveying water moisture. The wettability-gradient-induced membrane can be a contender for achieving a larger capacity that guides the conveyance of perspiration-driven water, out of the notorious dilemma of moisture transportation and opposite penetration [27–31].

The multilayer electrospun membrane displays the diode-like unidirectional transportation property due to the wettability gradient, making it a potential option for the fabrication of thermoregulatory textiles with superior PCE and water moisture-wicking capacity without reverse penetration. Apart from tailoring wettability, passive cooling technology can manage the skin microenvironment with zero-energy-consumption due to its intrinsic properties (e.g., structure and morphology) [32–36]. Specifically, selective matrices, e.g., polyvinylidene fluoride and polyurethane (PVDF and PU) are attractive materials for passive cooling technology owing to their effective emission in the region of MIR wavelength, which is devoted to its bond vibrations, such as PVDF (–CH_3_, –CH, C–C, CF_2_ bonds) and PU (–CH_2_, C–C, N–H, C = O bonds) [37–39]. Also, 2D transition metal carbides/nitrides, MXenes, are typically used as multifunctional fillers for thermoregulatory textiles in electrospun membranes owing to its excellent polar surface, intrinsic layered nanostructure, and metal-like thermal conductivity [40, 41]. Their hydrophilic surface, which results from the abundance of functional terminations (OH, O, and/or F), facilitates the formation of a strong interfacial interaction with nanofibers during the electrospinning process [42, 43]. The layered architectures of these functional MXene nanosheets with water-induced interlayer channels could equip the resulted fabrics with larger water moisture-wicking capabilities involved in evaporative cooling effect. Moreover, the layered structure of conductive MXene nanosheets permits interaction with polar polymer textiles without sacrificing its thermal conductivity, resulting in potential applications in thermoregulatory domains [44, 45]. Although MXene-based nanofibers and MXene-decorated electrospun membranes have been developed for various applications, e.g., supercapacitors, sensors, and personal heating management systems, however, MXene-engineered multilayer membranes with water moisture-wicking transportation for PCE have not yet been investigated [46–48].

Herein, this study attempts to present a feasible and effective strategy for designing a passive-evaporative cooling membrane to advance its thermophysiological comfort in personal cooling management. Specifically, we proposed a wettability-gradient-induced-diode (WGID) membrane composed of PVDF&PU nanofibers and MXene-engineered polyurethane membranes (PU@MXene), which imparts an efficient, localized cooling effect and the capacity to convey water moisture out without reverse penetration. Our design of the WGID membrane primarily benefits from the high MIR-emissivity of 96%, the thermal conductivity of 0.3349 W m^−1^ K^−1^, and the wettability-gradient of the integrated membrane, to provide an effective passive-evaporative cooling effect. On the one hand, the high-emissivity property and thermal conductivity of MXene-engineered electrospun membranes allow more heat dissipation along with its interwoven structure based on radiative- and conductive-controlled pathways, thereby obtaining a 1.5 °C higher cooling temperature in a “dry” state when compared to traditional cotton. On the other hand, the wettability gradient of electrospun membrane is capable of releasing the water moisture transportation and evaporative heat from the inner hydrophobic side to the outer hydrophilic side in a “wet” state, resulting in the increased cooling temperature by 7.1 °C than that of cotton counterpart. The developed WGID membrane with passive-evaporative cooling effect and moisture-wicking capability, leveraged on the MXene-engineered design, provides an innovative strategy for overcoming the performance limitations of traditional cotton toward personal cooling management.

## Experimental Section

### Materials

Polyurethane (PU) was supplied by Shanghai Huntsman Polyurethanes Specialties Co., Ltd., China. Dimethylformamide (DMF), Lithium Chloride (LiCl), and Sodium Hydroxide (NaOH, pp%) were purchased from Sinopharm Chemical Reagent Co., Ltd., China. Lithium fluoride (LiF, 99.9%) was obtained by Shanghai Macklin Biochemical Co., Ltd., China. Ethylalcohol (95%) was provided by Shanghai Aladdin Biochemical Co., Ltd., China. MXene-Ti_3_C_2_ powders were purchased from Jinlin 11 Technology Co., Ltd., China.

### Preparation of PVDF&PU Electrospun Membranes

To prepare the PVDF&PU solution, PVDF and PU were dissolved in the DMF solvent at 60 °C for 24 h (PVDF: PU: DMF = 10:10:80, mass ratio). The PVDF&PU membrane was obtained with uniform distribution of nanofibers. The electrospun parameters were set as follows: applied voltage, 15 kV; collector distance, 15 cm; syringe speed, 1.5 mL h^−1^.

### Fabrication of PVDF&PU/PU@MXene Membranes

The PU@MXene solutions for electrospinning technology were obtained by ultrasonic dispersion of MXene powder and dissolving PU in the DMF solvent at room temperature for 24 h. For the preparation of PU@MXene single-layer fibrous membranes, the weight ratios of PU, MXene, and DMF solvent was 18:2:80 and 16:4:80. The PU@MXene (10 wt%) fibers were uniformly deposited on the PVDF&PU membrane; then, the PU@MXene (20 wt%) fibers were further deposited on the before-obtained membrane; finally, a tri-layer membrane of PVDF&PU/PU@MXene(10%)/PU@ MXene(20%) was obtained. The electrospun parameters were set as follows: applied voltage, 15 kV; collector distance, 15 cm; syringe speed, 1.5 mL h^−1^.

### Synthesis of WGID Membranes

**Fig. 1:**
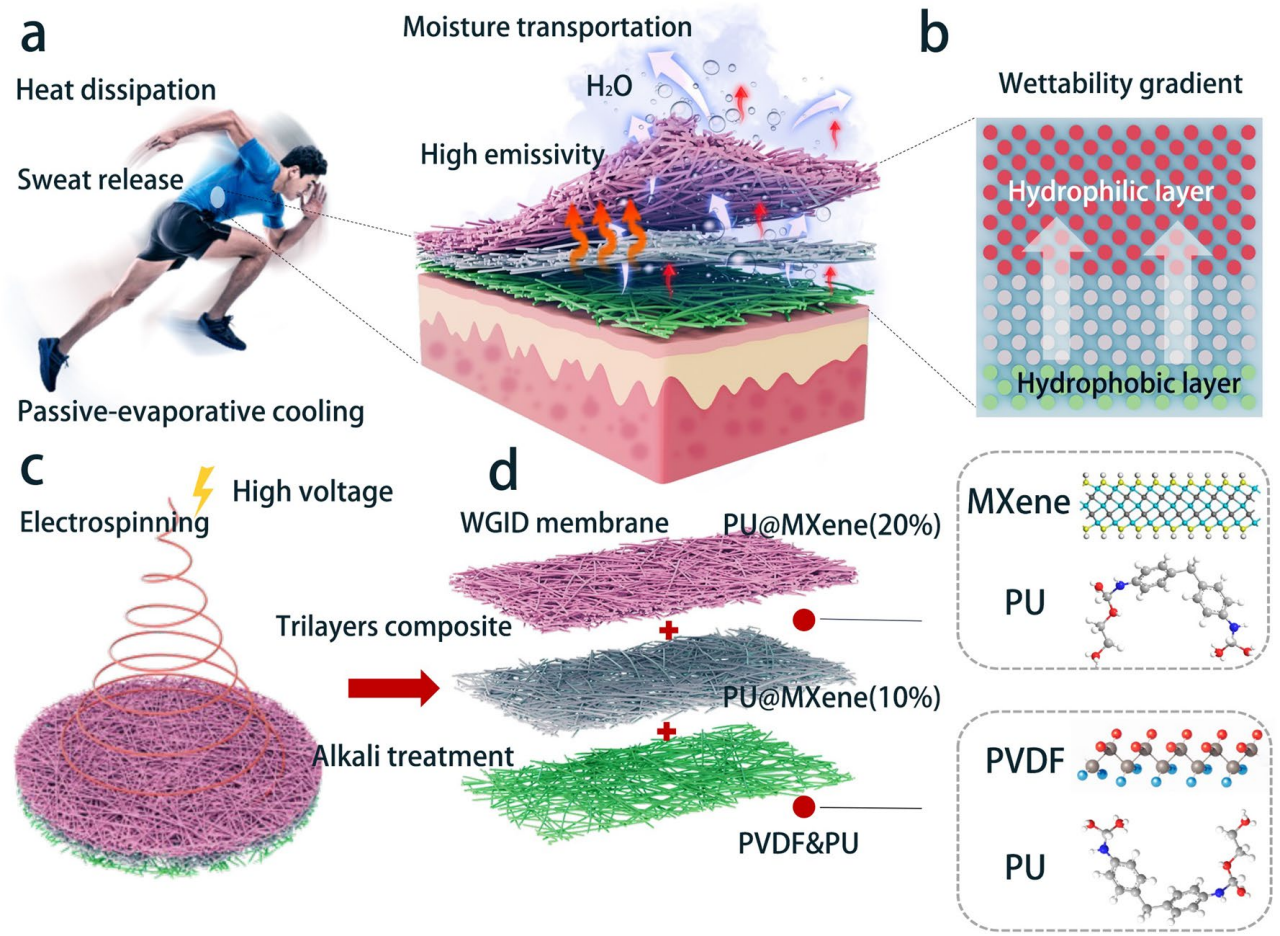
Schematic depicting the heat dissipation and sweat release process of **a** WGID membrane. **b** Schematic structure of WGID with wettability gradient property from hydrophobic to hydrophilic layer. **c** Fabrication process of WGID via electrospinning technology and alkali treatment. **d** Composition of WGID membrane

The as-electrospun tri-layer membranes were placed into NaOH solutions at room temperature for 15 min, with different concentrations of NaOH solution. As-obtained membranes with alkali treatment were then rinsed with deionized water and dried at 60 °C in a vacuum oven for 24 h. The tri-layer membranes with alkali treatment were immersed with various concentrations of NaOH solution (including 0, 0.25, 0.5, and 1 M). The resultant tri-layer membrane exhibits the wettability qualities that vary across its layers, enabling the controlled and unidirectional transport of perspiration toward the outer surface. The schematic diagram is presented in Fig. 1, illustrating the precise structure and composition of the WGID membrane.

### Characterizations of WGID Membranes

Transmission electron microscopy, (TEM, Talos F200S G2 TEM, United States) and scanning electron microscope (SEM, U8100 (Regulus8100), Hitachi, Japan) were used to acquire the surface and cross-section morphology of WGID membranes. The phase structure and elemental valence were characterized using X-ray diffraction analysis (XRD, D2 PHASER, Bruker AXS, Germany) and X-ray photoelectron spectrometer (XPS, Axis Ultra DLD, Kratos, United Kingdom). On a controlled thermoplate, thermal images were captured using infrared thermography (TESTO 871, Germany) with a size of 2.5 cm × 2.5 cm. Bond vibrations were confirmed using Fourier transform infrared spectroscopy (FTIR, Nicolet 6700, Thermo Scientific, USA). To determine the thermal oxidation resistance, a thermoanalyzer (TGA2, Mettler-Toledo, Switzerland) was used to execute thermogravimetric analysis. The hydrostatic pressure was obtained via the FX 3000 Hydro Tester. To exhibit thermal radiation behavior, an FTIR spectrometer (PerkinElmer's Spotlight 200i) equipped with an infrared integrating sphere was utilized. The water vapor transmission rate (WVTR) was analyzed using Haida International Equipment (Model-HD-E702-100–4, Dongguan, China) in accordance with ASTM E96. The weight of the membrane (10 cm × 10 cm) was measured every 5 min after 0.2 g of water was poured on it, which was conducted based on GB/T 21655.1 − 2008. The fluorescent green water droplets were landed on the WGID membrane to track the movement of fluorescent ink. Water contact angle (WCA, with 5 μL water droplet) on membranes was tested using a contact angle goniometer (Kino Industry Co., Ltd.). The thermal conductivity of the as-prepared membranes was determined using Thermo Labo II guarded heated plates. To ascertain the consistent thermal conductivity, the WGID membranes were strategically placed within the interleaf region of two plates. One plate was cooled to a temperature of 25 °C, while another plate was allowed to reach a temperature of 35 °C. The washing experiment involved agitating the WGID membrane in dynamic water at the room temperature and a rotational speed of 600 rpm min^−1^ for varying durations. The residual mass of the dry WGID membrane was measured at regular intervals of 2 h. The rubbing property was evaluated by subjecting the WGID membrane surface to a 200 g weight and performing a reciprocating friction motion of 5 cm in length.

## Results and Discussion

### Design, Fabrication and Characterization of WGID Membranes

Based on a wettability-gradient electrospun nanofibers with passive-evaporative cooling effect, water moisture-wicking without reverse penetration, as depicted in Fig. 1a, the WGID membrane can more effectively achieve heat dissipation and sweat release from the inner side (close to the human skin) to the outer side. The achieved result mostly involves in the high emissivity and thermal conductivity for heat loss, and the meta-structure with a wettability gradient for continuous water moisture transport (Fig. 1b). Furthermore, following the process of an alkali treatment to the tri-layered membrane, the wettability of each electrospun nanofiber was altered. Importantly, this change in wettability proved to be highly advantageous for achieving an efficient passive-evaporative cooling effect in a high humidity environment, without undesirable reverse penetration. This is a major advantage of the proposed construction for assuring the accessibility and effective transportation of water moisture with heat capacity. Figure 1c schematically depicts the synthesis of the WGID membrane with passive-evaporative cooling effect and moisture-wicking capacity. Further details are given in the Supplementary Information. Briefly, the multilayered WGID membranes were prepared via electrospinning technology followed by alkali treatment. The WGID membrane consists of a PVDF&PU hydrophobic layer, a PU@Mxene(10%) transport layer and a PU@Mxene(20%) hydrophilic layer, as exhibited in Fig. 1d. Thus, the tri-layered nanofibers produced a WGID membrane with a thickness of about 120 µm in Fig. S1, which could be served as a thermoregulatory material exhibiting an excellent passive-evaporative cooling effect with unidirectional water transportation property, due to the tailored radiative emissivity in the MIR region and asymmetric wettability of each layer.

**Fig. 2:**
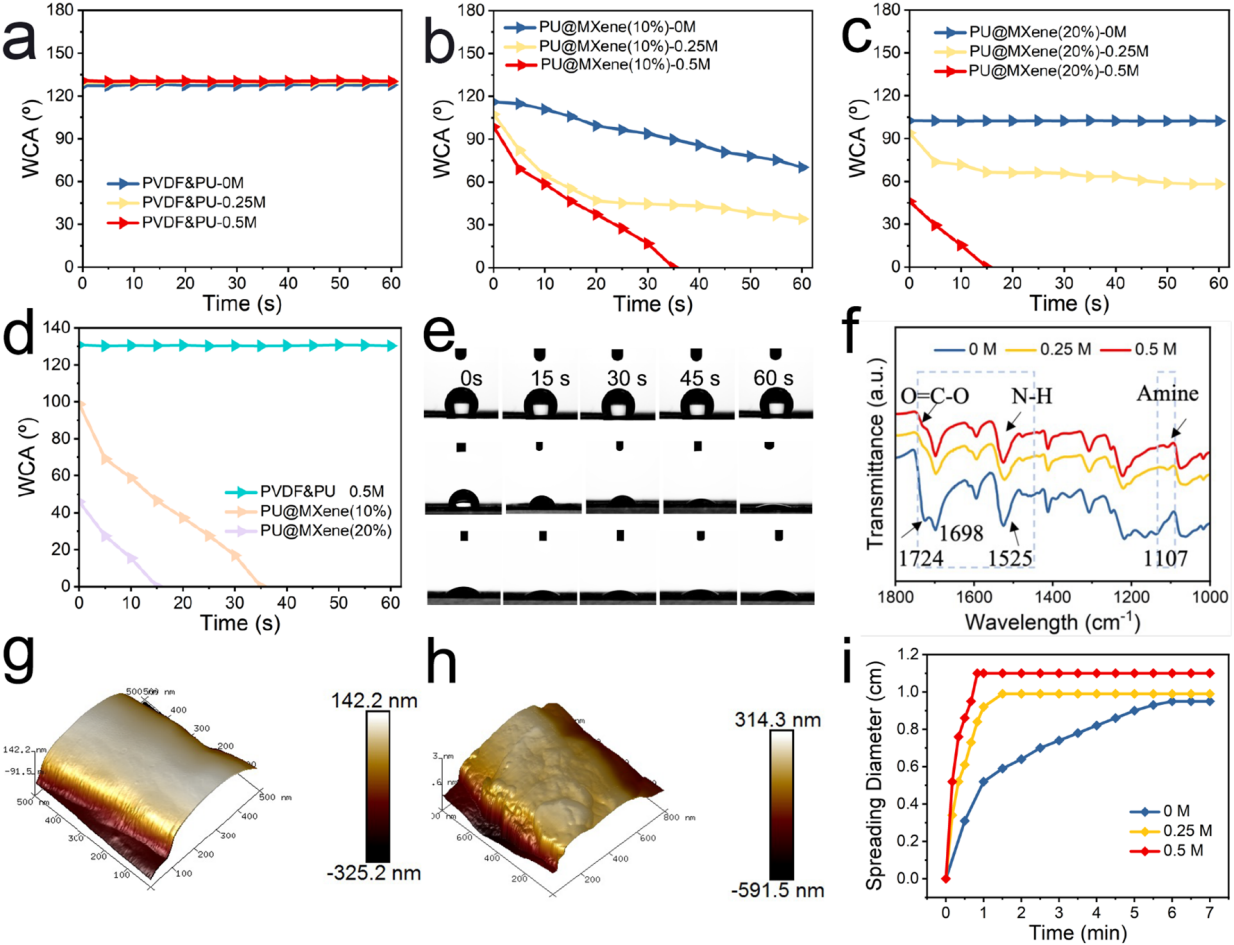
Curves of apparent water contact angle change of **a** PVDF&PU, **b** PU@MXene(10%), and **c** PU@MXene(20%) in different concentrations of NaOH solution (0 M, 0.25 M, 0.5 M). **d** Curves of apparent water contact angle change. **e** Optical photographs of water contact angle variations (from top to bottom) of PVDF&PU, PU@MXene(10%), and PU@MXene(20%) in 0.5 M NaOH solution. **f** FTIR of PU@MXene(20%) with alkali treatment in different concentration of NaOH solution. AFM images of **g** PVDF&PU, **h** PU@MXene(20%), and **i** spreading speed of PU@MXene(20%) in different concentrations of NaOH solution

In order to gain insight into the behavior of water moisture transportation for various electrospun membranes, hydroxyl groups, MXene mass, and alkali treatment duration are considered as factors to construct the initial wettability gradient. By modifying these parameters, we first fabricated electrospun membranes of PVDF&PU, PU@Mxene(10%), PU@MXene(20%) and PU@MXene(30%), further introduced carboxylate group (− COO) and amine group on the surface of electrospun nanofibers by immersing in various concentrations of NaOH solutions (0, 0.25, 0.5, and 1 M) for different times. The electrospinnability of PU@MXene(10%) and PU@MXene(20%) membranes led to their selection as the carriers of carboxylate and amine groups. When the mass ratio of MXene nanosheets was extensive to 30 wt% based on mass ratio of PU, aggregations of MXene were observed on the surface of as-electrospun membrane (Fig. S2a–c). As depicted in Fig. 2a, the alterations in the apparent water contact angle curves of PVDF&PU coming from alkali treatment with different concentrations for a duration of 5 min were found to be negligible, resulting in a hydrophobic layer with a substantial water contact angle of 129°. For PU@MXene(10%) membrane, its hydrophilicity was altered after the immersion in NaOH solution, which is endowed with improved hydrophilicity and enhanced wettability as the concentration of the solution increases. Specifically, the pristine PU@MXene(10%) membrane (without alkali treatment) exhibited underperformed wettability with the water contact angle from 115° to 80° in 1 min. And the alkali treatment membrane within 0.25 M NaOH possessed a better hydrophilicity along with 45° after 60 s. While the electrospun membrane (immersed in 0.5 M NaOH) displayed a dramatic drop in water contact angle from 101° to 0° within 35 s, indicating superior wettability in comparison to the membrane without alkali treatment in Fig. 2b.

As for PU@MXene(20%) electrospun membrane, it manifested the similar tendency as the electrospun membrane was soaked in a higher concentration, rendering facilitated wettability. The water contact angle of PU@MXene(20%) membrane changed remarkably from hydrophobicity to hydrophilicity, where the as-electrospun membrane without alkali treatment was hydrophobic with a value of 105°, and the outstanding hydrophilicity was obtained by achieving a fully wetted state within 15 s after alkali treatment with 0.5 M NaOH solution. When the concentration of NaOH solution was enlarged, the as-obtained membranes encountered damage to their interlocking structure in Fig. S2d–f, due to inadequate alkali resistance of PU polymer. Moreover, in the process of introducing carboxylate and amine groups, the immersion duration is a major design parameter that requires optimization. It should be noted that the as-electrospun membranes exhibited no essential change in hydrophilicity since the saturated carboxylate and amine group was introduced onto the surface of nanofibers, when the immersion period was extended to 10 min from 5 min (Fig. S3). In detail, the water contact angle of PU@MXene(20%) was decreased from 43° to 0° in 12 s, exhibiting an enhancement of 3 s than that of 5 min treatment counterpart. Correspondingly, the optical images of each layer were clearly demonstrated the well-wetted gradient with the consistent outcomes of water contact angle changes (Fig. S4).

To strike a balance between wettability-gradient and cost-effectiveness, the WGID membrane was obtained by an alkali treatment of 0.5 M NaOH solution for 5 min, which was deemed a suitable design tradeoff and selected for subsequent measurements. Both curves of apparent water contact angle change and optical photographs, they exhibited a great wettability gradient of the WGID membrane with 0.5 M NaOH solution for 5 min. Whereas, other conditions might result in over-wettability or inferior wettability, as shown in Figs. 2d, e and S5. To further demonstrate the enhanced wettability of MXene-engineered electrospun membranes treated with alkali solution, their surface chemical structure was measured by FTIR. With increasing the concentration of NaOH solution, the PU@MXene electrospun membranes exhibited the desired hydrophilicity due to the existence of hydrophilic carboxylate groups (-COO^−^) and amide groups in Fig. 2f [49, 50]. An improved ability of spreading speed (Fig. 2i) could be obtained, ascribed to rougher surface than that of electrospun membranes without decoration of MXene nanosheets, as displayed in Figs. 2g, h and S6. The pristine PU@MXene(20%) membrane exhibited a hydrophobic property, however, it transformed into a hydrophilic surface after being treated with alkali solution, manifesting that the micro-/nanostructured roughness of the surface can make hydrophobic surfaces more hydrophobic and hydrophilic surfaces more hydrophilic [51, 52].

**Fig. 3:**
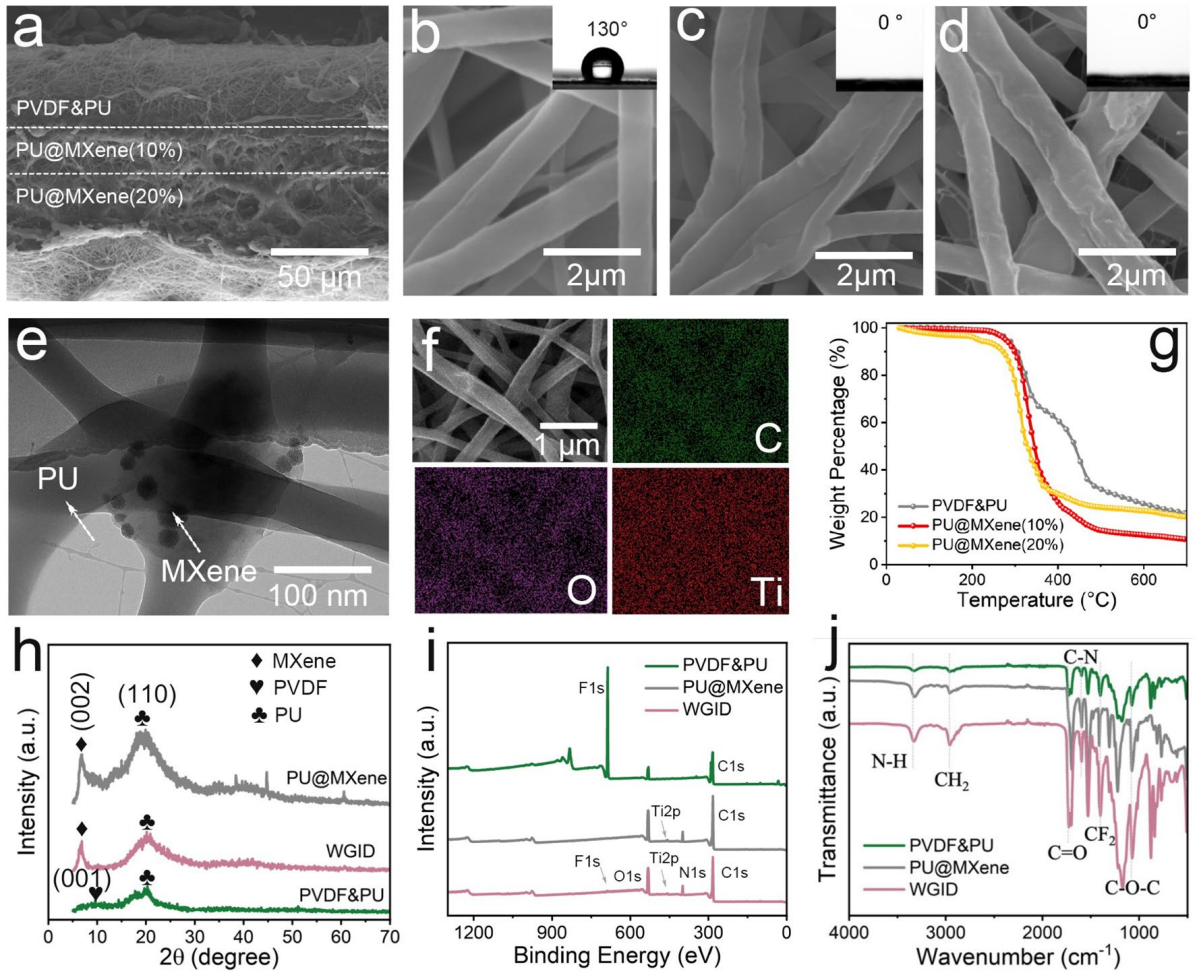
Cross-sectional SEM image of WGID. SEM images (inset of optical photographs) of **a** WGID membrane of **b** hydrophobic layer, **c** transport layer and **d** hydrophilic layer. **e** TEM image of PU@MXene electrospun membrane. **f** SEM and element mappings of WGID (hydrophilic side). **g** TGA curves. **h** XRD, **i** XPS, and **j** FTIR of PVDF&&PU, PU@MXene(20%), and WGID, respectively

The microstructure of electrospun MXene-engineered membranes was observed via the SEM and TEM. The cross-section morphology of the WGID membrane is shown in Fig. 3a, revealing that the proposed WGID membrane was consisted of a typical tri-layered structure on an electrospun membrane including PVDF&PU, PU@MXene(10%) and PU@MXene(20%). The uniform nanofibers of the WGID membrane could be observed with the inset of optical photographs in Fig. 3b–d, as well as the fiber diameter and pore size distribution as exhibited in Fig. S7. The electrospun membrane possessed a polymer–MXene skeleton, as shown by TEM images (Figs. 3e and S8). Also, the elements of demonstrated by EDS elemental mapping revealed that C, O, and Ti were uniformly distributed on the surface of the WGID sample (Fig. 3f), indicating that MXene is successfully incorporated into the polymeric matrix. The TGA curve numerically demonstrated the calcined samples of the PU@MXene electrospun membranes with MXene nanosheets loadings of 10 and 20 wt%, respectively, and PVDF&PU electrospun membranes with the result of 20 wt%, as exhibited in Figs. 3g and S9. Moreover, the XRD pattern of the as-electrospun membranes is displayed in Fig. 3h, which illustrates the structure of PVDF&PU, PU@MXene(20%), and the WGID membrane. The distinct peaks at 6.9° indicated a MXene lattice structure (002) packed inside the polymer–MXene skeleton. Also, the characteristic peak of PVDF&PU was located at 19.8° with lattice structure of (110) [37, 41]. To investigate the binding status of the elements, the chemical compositions of all as-electrospun membranes were characterized by XPS. As shown in Figs. 3i and S10, the Ti 2*p* peak was appeared in PU@MXene and the WGID electrospun membranes, assigned to Ti 2*p* 1/2, Ti 2*p* 3/2 with the corresponding binding energies of 463.73 and 458.28 eV, respectively, Ti − C with a value of 454.61 eV was also detected [43, 53]. Also, in contrast to the FTIR spectrum of PVDF&PU (Fig. 3j), the emerging absorption peaks at 3327 and 1724 cm^−1^ belong to the characteristic N–H stretching vibration and urethane bond of MXene-engineered electrospun membranes, respectively [37, 54]. As a result, we successfully fabricated a MXene-engineered WGID membrane for PCE which, as subsequently shown, rendered a tri-layered electrospun membrane.

### Unidirectional Moisture Transportation Behavior of WGID Membranes

**Fig. 4:**
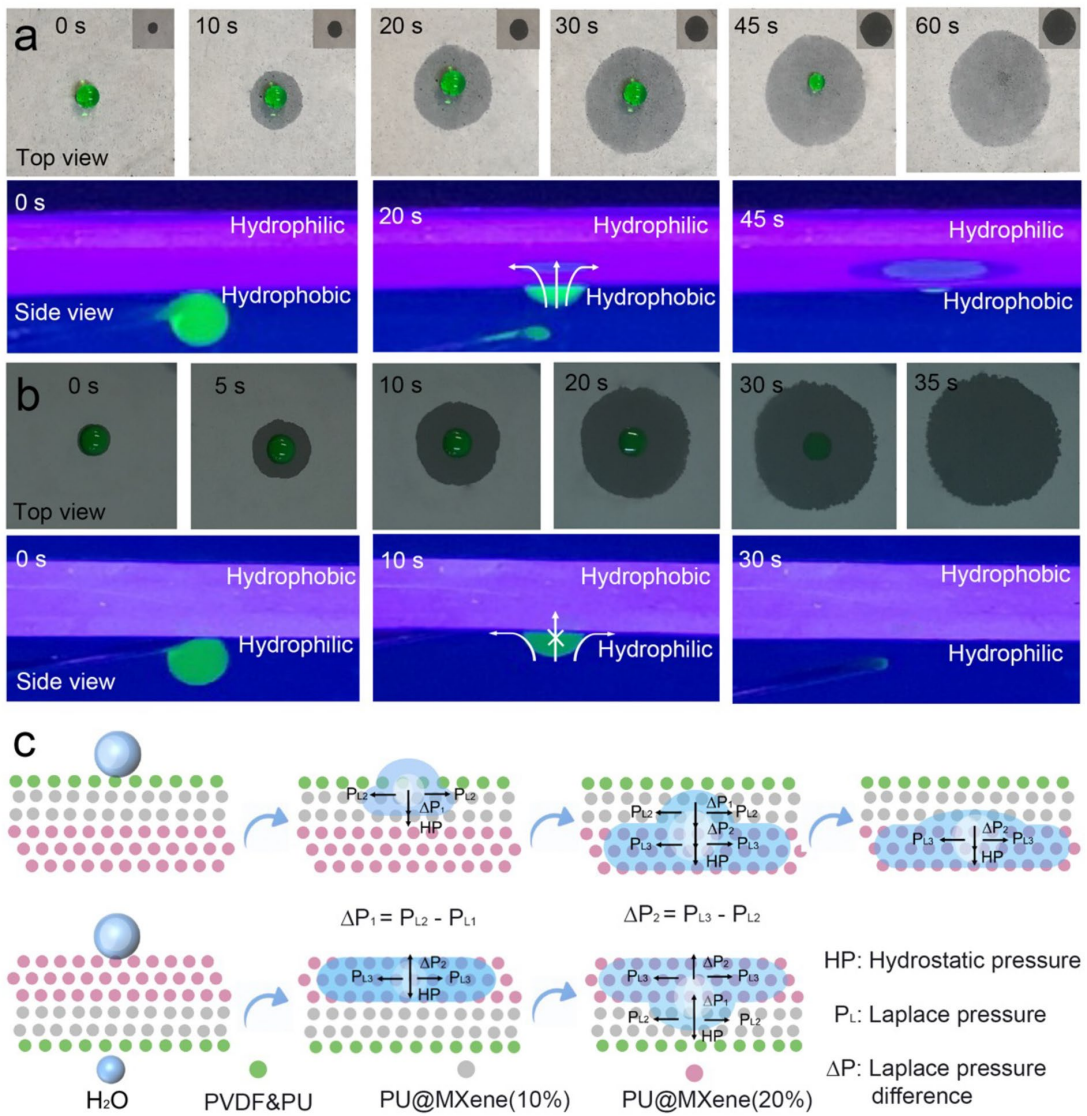
Water moisture transportation mechanism of WGID. Wetting behavior (green water droplets, 50 µL) of **a** PVDF&PU and **b** PU@MXene-(20%) layers of the photographs of top view and the fluorescent images of side view, respectively. **c** Schematic illustration of the simplified model explaining the water moisture transportation of WGID

The unidirectional water transportation behavior on the WGID membrane was confirmed by dripping a water droplet (50 μL) on two sides. Based on the foundation, the inner side (touch to the human skin) needs to permit excessive water moisture to escape spontaneously, eliminating sticky and uncomfortable feelings [27]. The contacted side is placed vertically into the water surface, mimicking the scenario of human skin, to highlight the differential more vividly in wettability between the two sides of the WGID membrane. As shown in Fig. 4a (top view), the water droplet could maintain a spherical shape, the wetting area was changed gradually, and the backside of WGID simultaneously was gotten wetting, as observed in the inserted photographs, illustrating that the WGID membrane could penetrate and then spread from the PVDF&PU side to the PU@MXene(20%) side. However, once the droplet was dripped on the outer hydrophilic side under the identical circumstances, the water droplets immediately collapsed and spread on the hydrophilic side in Fig. 4b (top view). After full spreading, the wetness zone was only observed on the inner hydrophilic side where the water droplets were deposited, whereas the wetting region on the opposite side was not observed (Fig. S11), manifesting that there was no penetration from the PU@MXene(20%) side to the PVDF&PU side. The observed phenomena exhibited consistent behavior in terms of the spreading diameter on both the hydrophobic and hydrophilic sides of the WGID membrane as depicted in Fig. S12, which illustrated its excellent performance of unidirectional moisture transport. Further demonstration of the unidirectional water moisture transportation of the WGID membrane, the anti-gravity experiment, delivering the fluorescent green water droplets exposed to the UV radiation (λ = 365 nm) upward to contact the inner hydrophobic side (Fig. 4a, side view, and Movie S1) and outer hydrophilic side (Fig. 4b, side view, and Movie S2), respectively, was conducted. When the fluorescent green water droplet came into touch with the PVDF&PU hydrophobic side, the water droplet was penetrated from the bottom and then gradually spread to the top. Meanwhile, once the fluorescent green water droplet was put on the hydrophilic surface of PU@MXene(20%), the water droplet was blocked and only distributed on that contacted side, with no penetration trace observed. The hydrophilicity-to-hydrophobicity exhibited an impediment to water penetration, indicating that the WGID membrane with a gradient wettability possessed a diode-like feature and revealing desirable capacity of unidirectional water transport.

In general, when the water droplets encounter the fiber membranes, they enter the interfiber capillary channels between the fibers and are involved in the Laplace pressure (P_L_), which is calculated as the following equation [55]:

1 $${P}_{L}=\frac{4\gamma {\text{cos}}\theta }{r}$$

where $\gamma ,\theta$, and *r* represent the liquid–gas surface tension, the contact angle on the surface of the fibers, and the pore diameter of the nanofibers, respectively. Typically, the nanofibers membrane could be wetted with capillary force in the interfiber channels (WCA < 90°), but not with hydrophobic force (WCA > 90°). The direction of the capillary force is determined by the negative or positive outcome of the *P*_L_. Since the contact angles of the WGID membrane are asymmetric, the *P*_L_ results of water droplets in the two capillaries are different. At the interface between the PVDF&PU and PU@MXene(10%) two layers, the water droplet is subjected to two Laplace pressures (e.g., *P*_L1_ and *P*_L2_), with the resultant force being the Laplace pressure difference ($\Delta {P}_{1})$ in the upward trend [5, 55].

2 $$\Delta {P}_{1}={P}_{L2}-{P}_{L1}$$

Furthermore, the Laplace pressure (related to *P*_L2_ and *P*_L3_) of the liquid at the interface between the PU@MXene(10%) and PU@MXene(20%) two layers, the Laplace pressure difference is Δ*P*_2_ [10].

3 $$\Delta {P}_{2}={P}_{L3}-{P}_{L2}$$

Upon entering the PVDF&PU hydrophobic layer, the water droplet is subjected to an upward force, demonstrating the unidirectional, irreversible, and even anti-gravity moisture transport. Once the water droplet impacts on the PU@ MXene(20%) hydrophilic surface of the WGID membrane, it rapidly spreads due to the existence of the strong Laplace pressure and reverse hydrostatic pressure, rendering the accumulation of water moisture on the hydrophilic surface. Thus, the asymmetric wicking property of tri-layered membranes can be attained by tailoring the wettability of each individual layer, resulting in unidirectional water transport in the electrospun nanofibers.

A simple model could be used for exploring the unidirectional water transportation mechanism of the WGID membrane based on the obtained outcomes, as illustrated in Fig. 4c. When the water droplet was landed on the WGID membrane, contacted with hydrophobic side first, it maintained in a state with two reverse pressures, including Laplace pressures (*P*_L_) and hydrostatic pressure (*HP*) [32, 55, 56]. Where the *HP*, defined as “the pressure exerted by gravity on a fluid in equilibrium” is height-dependent of water droplet. The water droplets may typically infiltrate from the hydrophobic layer (PVDF&PU, represented by green balls) to the transport layer (PU@MXene(10%), depicted by gray balls). Then, the Laplace pressure difference between of the hydrophobic layer and transport layer ($\Delta {P}_{1}$), along with *HP*, allowed the water to spread across the transport layer. Furthermore, the water moisture would move forward and propagate throughout the hydrophilic layer (PU@MXene(20%), indicated by pink balls) with assistance of its Laplace pressure difference ($\Delta {P}_{2}$). As a result of the pressure difference, the accumulated water is promptly conveyed from the hydrophobic layer to the hydrophilic layer, without reverse transportation. Also, the water moisture contacted on the hydrophilic layer and quickly spreading out, due to the existence of Laplace pressure (*P*_L3_) on that surface. When the water moisture reached to the intermediate layer, its late penetration slowed owing to the opposite direction of pressure difference, which was detrimental to water moisture conveyance. Moreover, the hydrophobic side might further hinder the penetration of water moisture by exerting a larger reverse hydrophobic force. With the same direction of penetration, the pressure difference, had a substantial effect on the transportation of water, culminating in the storage of water moisture in the outer hydrophilic layer [38, 56].

**Fig. 5:**
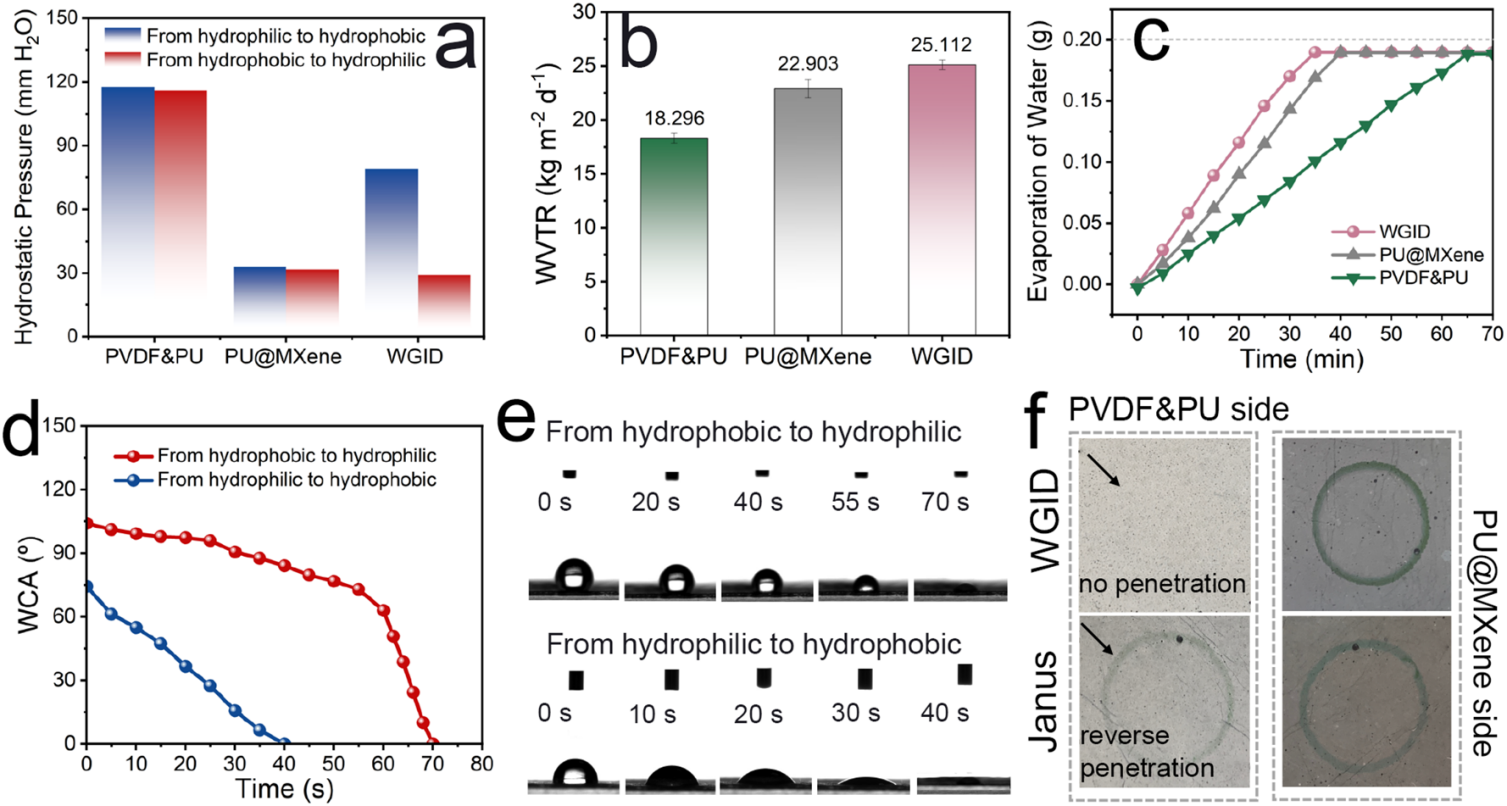
Hydrostatic **a** pressure, **b** WVTR, and **c** Evaporation of water of PVDF&PU, PU@MXene(20%), and WGID membranes. **d** Curve of the water contact angle and **e** optical photographs of water contact angle variations of WGID from hydrophobic and hydrophilic two sides. **f** Photographs of WGID and Janus membranes of PVDF&PU hydrophobic side and PU@MXene(20%) hydrophilic side

To demonstrate the unidirectional water moisture transportation through the electrospun membranes, the hydrostatic pressures from the PVDF&PU layer (hydrophobic side) and the PU@MXene layer (hydrophilic side) were measured (Figs. 5a and S13). Obviously, both sides of the PU@MXene membrane demonstrated a low hydrostatic pressure with the value of 29 mm H_2_O, where the water began to penetrate through the electrospun membrane. The hydrophobic layer exhibited a higher hydrostatic pressure of roughly 118 mm H_2_O on both sides, indicating that it effectively blocks the penetration of water moisture, and fails to unidirectional permeability. On the contrary, the hydrostatic pressures on the hydrophobic and hydrophilic sides of the WGID membrane are 35 and 83 mm H_2_O, respectively, indicating a similar property to that of an electronic diode. Moreover, the one-way water transportation capacity of the WGID membrane was superior compared with Janus membrane (PVDF&PU/PU@MXene(20%)) due to a larger difference in hydrostatic pressure between two sides. Furthermore, the WVTR results are good agreement with the hydrostatic pressure. The WGID membrane could exhibit the maximum WVTR of 25.112 kg m^−2^ d^−1^ due to the continuous water moisture transportation, in comparison to the PVDF&PU of 18.296 kg m^−2^ d^−1^, PU@MXene(20%) of 22.903 kg m^−2^ d^−1^, and Janus membrane of 24.104 kg m^−2^ d^−1^, as shown in Figs. 5b and S14. Also, the WGID membrane displayed a dramatically increased speed of water evaporation in the proper direction (from hydrophobic side to hydrophilic side) in Fig. 5c. The water contact angle curve and the optical images of WGID membrane were exhibited from two sides in Fig. 5d, e. When the droplet was loaded from the hydrophobic side, the water contact angle was at 105°, and then gradually decreased in the first 50 s, further promptly reduced in the last 20 s, to achieve fully wetted. Meanwhile, the full wettability was achieved within 38 s when the droplet was brought into touch with a hydrophilic surface. These results indicate that the water moisture can be pumped from the hydrophobic side (generally, close to the skin surface) to hydrophilic side, and finally spread out on the hydrophilic surface, devoted to the wettability gradient of the multilayered electrospun nanofibers. In order to provide additional evidence for the moisture-wicking capability of the WGID membrane, it was compared to the Janus membranes. The results suggested that the WGID membrane might be out of the dilemma of moisture-wicking property and reverse penetration, with a short duration within 70 s being fully wetted and without reverse penetration, as shown in Figs. 5e and S15. The disparity in wettability led to the uninterrupted diffusion of water moisture across the electrospun membranes, preventing reverse penetration, hence accelerating moisture-wicking transportation and evaporation.

### Passive-Evaporative Cooling Effect of WGID Membranes

**Fig. 6:**
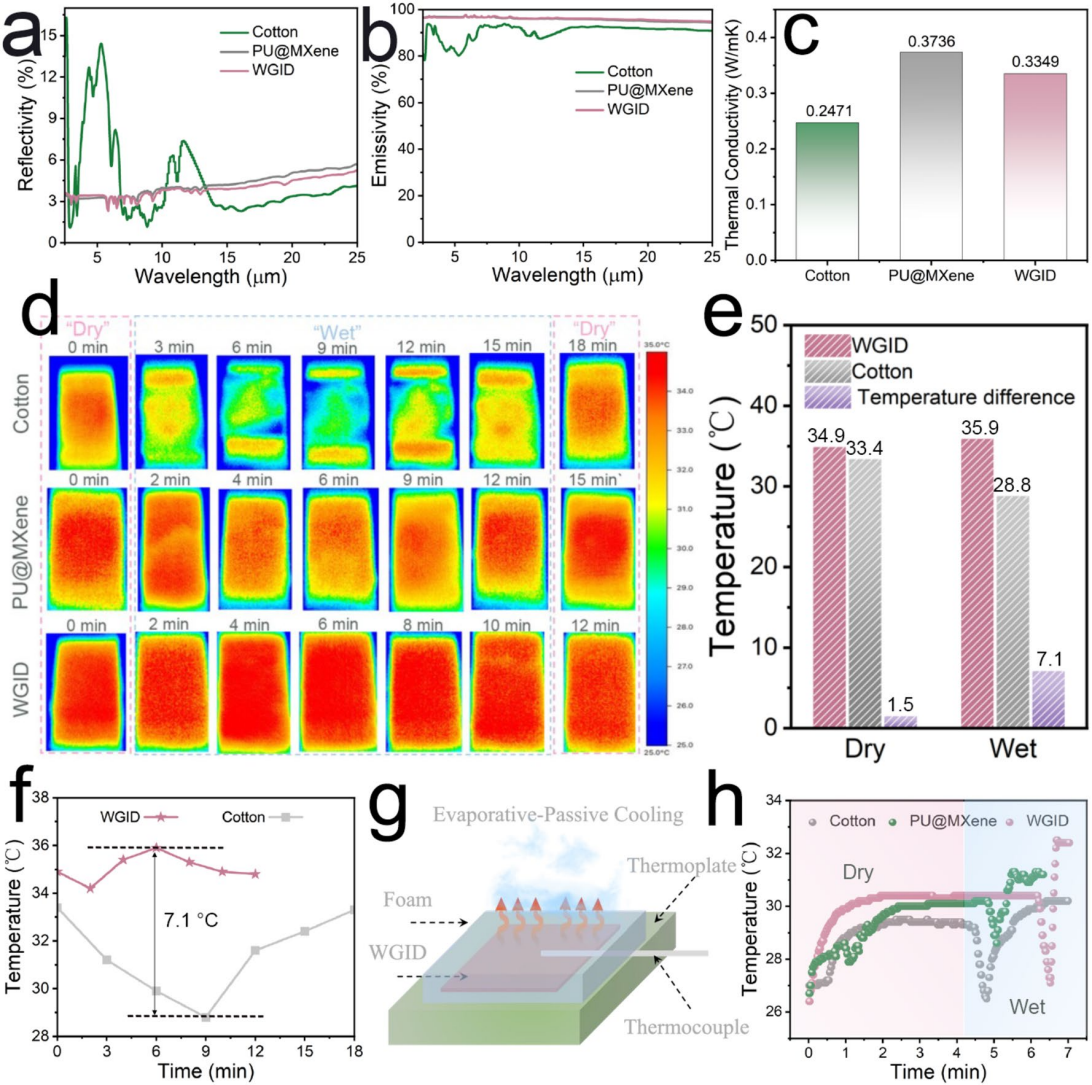
Reflectivity **a** emissivity, **b** thermal conductivity, of **c** traditional cotton, PU@MXene(20%), and WGID. The record of infrared images from dry state to wet state for traditional cotton, PU@MXene(20%) and WGID membrane. **d** Infrared thermal images, **e** temperature comparison, **f** curve of temperature change. **g** Proof-of-concept experiments for PCE. **h** Real-time temperature record of samples

In addition to the moisture-wicking property, the thermal heat necessitates to dissipate as much as possible for passive-evaporative cooling performance to achieve synergistic effect [5]. The better PCE of these samples has been proven by the characterizations of FTIR (with a gold integrated sphere) and Thermo Labo II, along with a comparison to the properties of traditional apparel. The MXene-engineered electrospun membranes displayed the high-emissivity and low-reflectivity in the MIR wavelength (Figs. 6a, b and S16), these values are derived via the equation of thermal radiation [5]:

4 $$\varepsilon =1-\rho -\tau$$

The variables *ε, ρ, *and *τ* are used to denote the values of emissivity, reflectivity, and transmissivity, correspondingly. The PU@MXene and WGID membranes possess an average emissivity of 96.19% and 96.40%, respectively, in the MIR wavelength. And the low reflectivity is another important factor that allows more heat dissipation from human body, with specific results of 3.80% for PU@MXene(20%) membrane, and 3.58% for the WGID membrane, respectively. In contrast, the traditional cotton exhibited an emissivity of 89.57% in the range of 8 to 13 µm, and a low reflectivity of 4.28%, overlapping with the radiative region of human body.

Furthermore, thermal conductance is an essential channel for heat dissipation, since it is the only way to transfer heat away inside textiles [3]. The thermal conductivity is determined by employing the heat input to the heated plate and the thickness of the sample, as outlined in the following equation [9]:

5 $${\text{k}} =\frac{\hbox{w}\cdot\hbox{D}}{\hbox{A}\cdot\Delta\hbox{T}_{0}}$$

where the thermal conductivity is denoted by k, the heat loss is represented by W, the thickness of the sample is indicated by D, the measurement area is denoted by A, and the temperature differential is represented by ∆T_0_, which is typically assumed to be 10 °C. The thermal conductivity of traditional cotton with the value of 0.2471 W m^−1^ K^−1^ was inferior to that the MXene-engineered electrospun membranes, e.g., PU@MXene(20%) of 0.3736 W m^−1^ K^−1^ and WGID membrane of 0.3349 W m^−1^ K^−1^ (Fig. 6c). As depicted in Fig. S17, the thermal conductivity of electrospun membranes increased as more MXene nanosheets were embedded, whereas the PVDF polymer incorporation had little effect on thermal conductivity. Furthermore, the infrared images were obtained using a thermal camera, which involved in positioning the samples on the upper surface of a hotplate maintained at a consistent temperature of 35 °C. In addition, a quantity of 0.05 g of water was applied onto the hydrophobic surface of the samples (with the size of 2.5 cm × 2.5 cm). The resulting variations in surface temperature on the outer layers were subsequently monitored and documented during the process of spontaneous drying, as illustrated in Fig. 6d. The observed surface temperature of traditional cotton was range from 28.8 to 33.4 °C for 18 min, which was lower than the PU@MXene and the WGID membranes, 31.6 to 34.3 °C in 15 min and 34.6 to 35.9 °C in 12 min, respectively, as shown in Figs. 6f and S18. Obviously, the as-obtained WGID membrane enabled to achieve a cooling temperature of 1.5 °C in a dry state, and 7.1 °C in a wet state compared to cotton (Fig. 6e). In the condition of dry environment, basically, the MXene-engineered membranes could achieve a higher surface temperature, devoted to the synergistic effect of radiative and conductive heat dissipation. When the environment is changed (from “dry” to “wet” state), cotton and PU@MXene membrane exhibited a propensity to grow following a reduction, while the temperature of WGID membrane climbs first and then declines gradually. These results were ascribed to the discrepancy of its inner hydrophilicity, the hydrophilic inner side of cotton and PU@MXene could facilitate the spreading of water molecules, resulting in the lock of water molecules and thermal energy generated by infrared radiation. Meanwhile, the WGID membrane enables to expedite the transportation of moisture from inner hydrophobic surface to the outer hydrophilic surface, thus possessing a passive-evaporative cooling effect. Furthermore, the moisture and heat management capabilities of the WGID membrane exhibited negligible changes following a continuous 12 h washing process and being subjected to 10 repetitions of rubbing with a weighted object. This observation highlights that the WGID membrane exhibits the exceptional resistance to washing (Fig. S19) and the capacity to withstand abrasion (Fig. S20).

Moreover, proof-of-concept experiments with different samples were conducted to record its real-time temperature to illustrate the passive-evaporative cooling performance (from “dry” to “wet” state). For this purpose, a thermoplate experiment was first utilized, with the final temperature set to 35.0 °C to simulate the temperature of human skin, as shown in Fig. 6g. When the electrospun membranes with the same size as infrared imaging experiments were placed onto the heating thermoplate, the temperature of the sample began to increase and eventually reached thermal equilibrium with a constant surface temperature, displayed in Fig. 6h. Obviously, the MXene-engineered membranes illustrated a higher surface temperature than traditional cotton, due to the high-emissivity and low-reflectivity in the MIR wavelength and superior thermal conductivity, which is in excellent accord with the results of infrared images. When the samples are in a wet state, the introduce of water results in a rapid fall in surface temperature, manifesting that all samples possess the capacity of evaporative cooling. The WGID one undergoes a fast transition period (less than 15 s) to efficiently achieve PCE, attributed to quickly moisture-wicking transportation and synergistic effect of passive-evaporative cooling. As for PU@MXene membrane and traditional clothing, they need take a longer time to attain the terminal equilibrium, with the resultants of 32 s and over 1.5 min, respectively. Based on previously obtained outcomes, the WGID membrane exhibited an effective effect of passive-evaporative cooling in conjunction with moisture-wicking transportation, leading to non-hampered heat dissipation for personal cooling management.

## Conclusion

This paper presents a MXene-engineered design for tri-layered electrospun membranes with outstanding passive-evaporative cooling effect. Benefiting from synergistically unidirectional water moisture transportation and efficient heat dissipation (both radiation- and conductance-controlled pathways), the WGID membrane exhibited an effective passive-evaporative cooling effect, thus providing thermophysiological comfort. As a result, the obtained WGID membrane enabled to achieve a cooling temperature of 1.5 °C in a “dry” state, and 7.1 °C in a “wet” state, due to its high emissivity of 96.40% in the MIR wavelength, superb thermal conductivity of 0.3349 W m^−1^ K^−1^ and unidirectional moisture transportation, compared to traditional cotton of bidirectional transportation. This work, the concept of wettability-gradient multilayered membranes, sheds light on the further development of MXene-engineered thermoregulatory textiles with both efficient cooling effect and unidirectional moisture transportation capacity.

## Supplementary Information

Below is the link to the electronic supplementary material.

Supplementary file1 (MOV 6143 kb)

Supplementary file2 (MOV 6382 kb)

Supplementary file3 (PDF 1721 kb)
