# Supplementary material for: Wettability Gradient-Induced Diode: MXene-Engineered Membrane for Passive-Evaporative Cooling
Source: Nanomicro Lett. 2024 Mar 21;16:159. doi: 10.1007/s40820-024-01359-8 (PMC10957859; doi:10.1007/s40820-024-01359-8)
Supplement: Supplementary file 3 — Supplementary file3 (PDF 1721 kb) [file 40820_2024_1359_MOESM3_ESM.pdf]

Supporting Information for

Wettability Gradient Induced Diode: MXene-Engineered Membrane for Passive-Evaporative Cooling

Leqi Lei[1], Shuo Meng[1], Yifan Si[1], Shuo Shi[1], Hanbai Wu[1], Jieqiong Yang[1], Jinlian Hu[1, *]

[1]Department of Biomedical Engineering, City University of Hong Kong, 999077, Hong Kong S. A. R, China

*Corresponding author. E-mail: jinliahu@cityu.edu.hk (Jinlian Hu)

Supplementary Figures

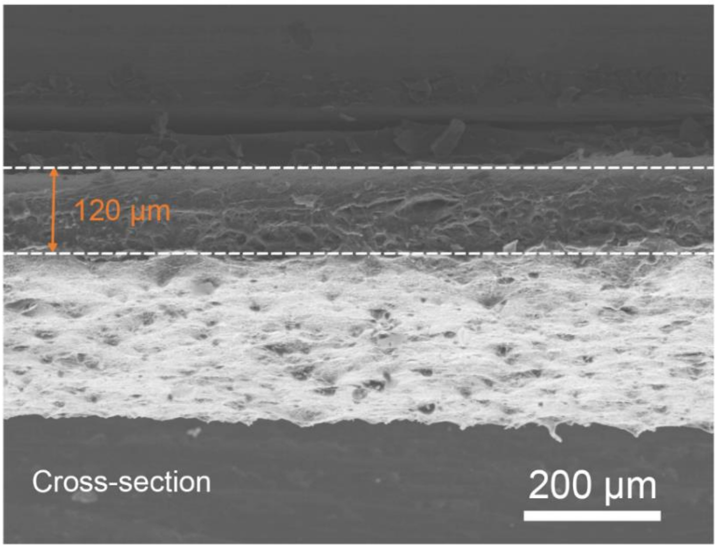

Fig. S1 Cross-section SEM image of WGID membrane

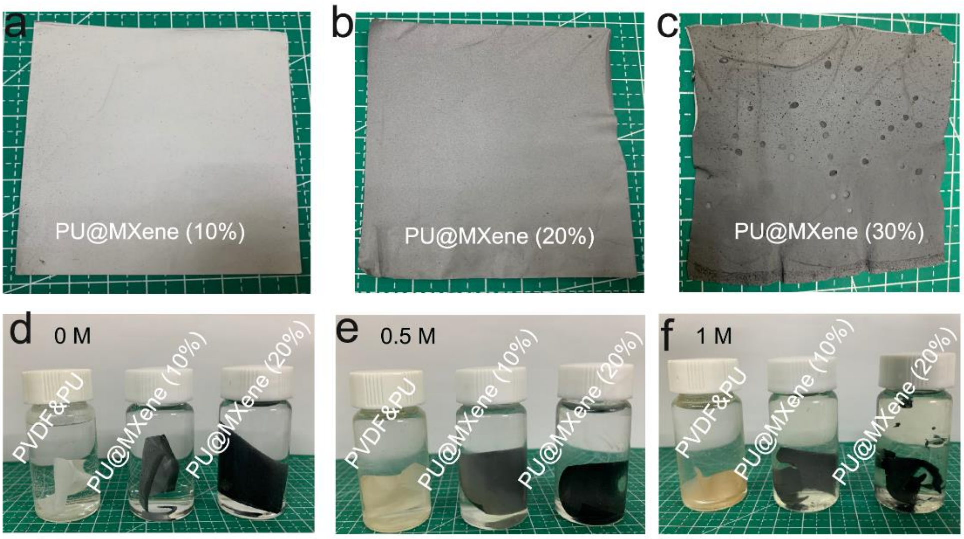

Fig. S2 Photographs of **a** PU@MXene(10%) membrane, **b** PU@MXene(20%) membrane, and **c** PU@MXene(30%) membrane. Photographs of samples for alkali treatment with **d** 0 M, **e** 0.5 M, and **f** 1 M NaOH solution

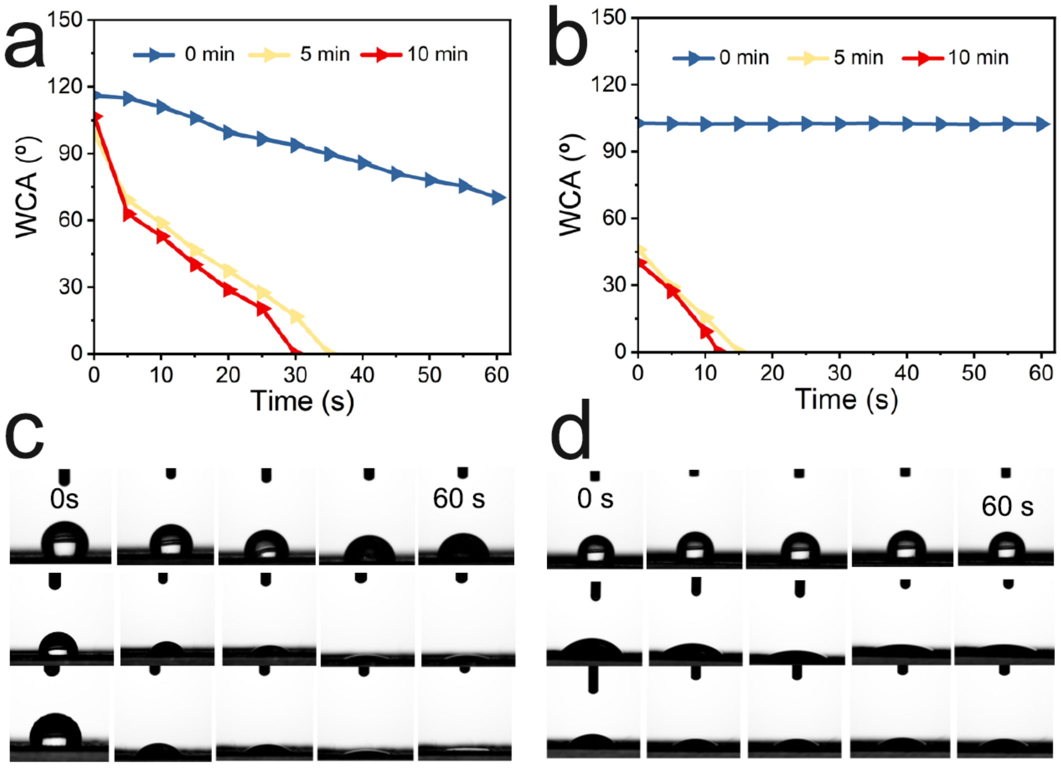

Fig. S3 Curves of apparent water contact angle change **a** PU@MXene(10%) and **b** PU@MXene(20%). Optical photos **c** PU@MXene(10%) and **d** PU@MXene(20%) in 0.5 M NaOH solution from top to bottom 0 min, 5 mins, and 10 mins

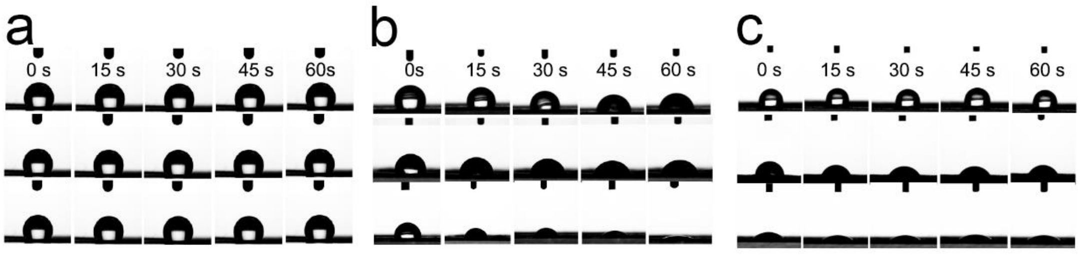

Fig. S4 Optical photos **a** PVDF&PU, **b** PU@MXene(10%), and **c** PU@MXene(20%) with different concentration from top to bottom 0 M, 0.25 M, and 0.5M for 5 mins of alkali treatment

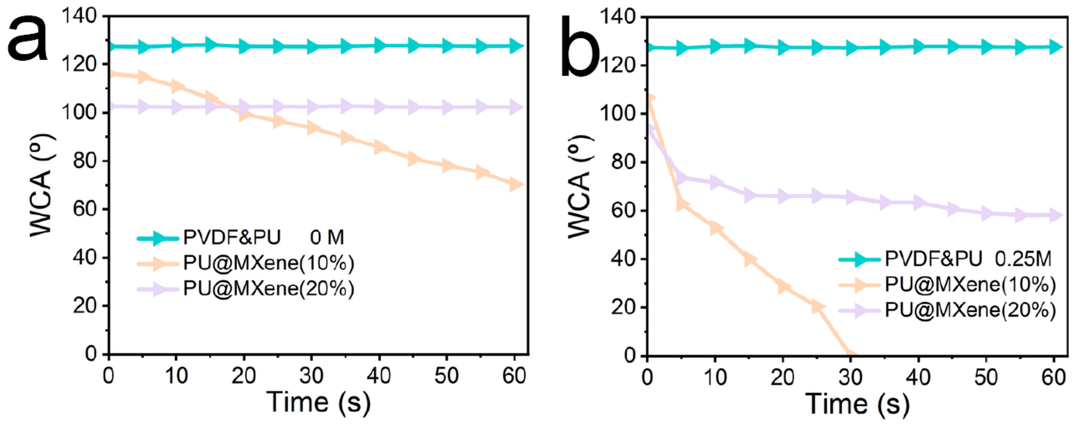

Fig. S5 Curves of apparent water contact angle change of PVDF&PU, PU@MXene(10%) and PU@MXene(20%) for different concentration of alkali treatment. **a** 0.25 M and **b** 0.5 M

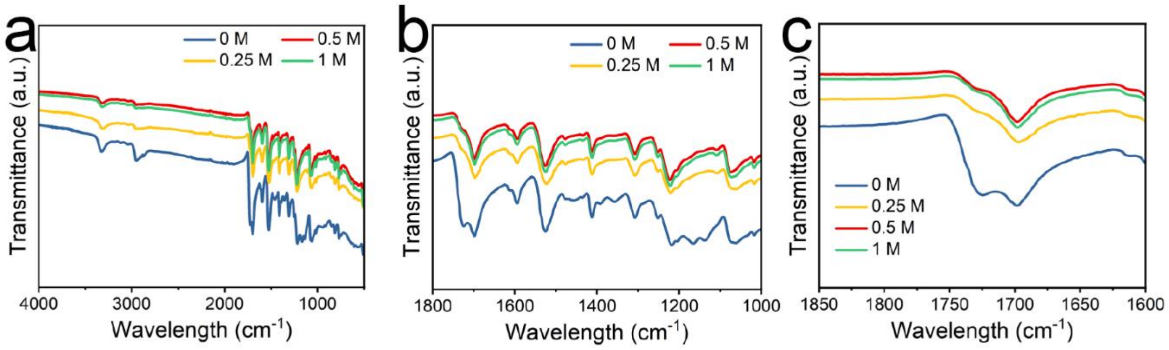

Fig. S6 FTIR of PU@MXene(20%) with alkali treatment in different concentration of NaOH solution. **a** 400-4000 cm^{-1}, **b** 1000-1800 cm^{-1}, **c** 1600-1850 cm^{-1}

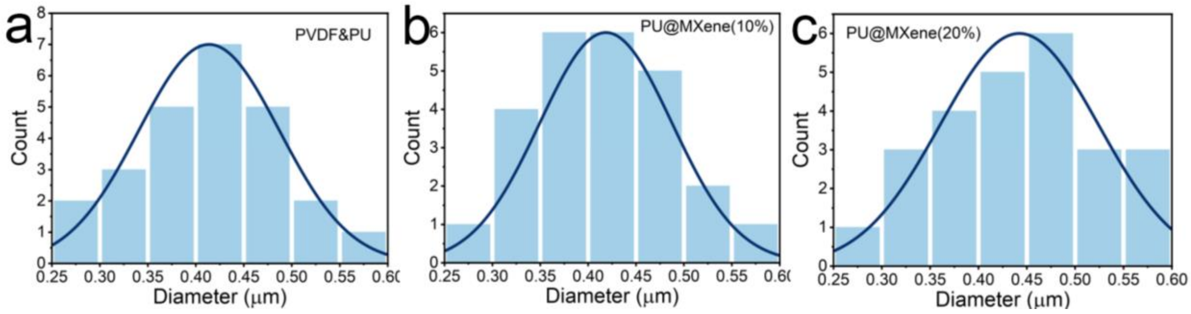

Fig. S7 Fiber diameter distribution of **a** PVDF&PU, **b** PU@MXene(10%), and **c** PU@MXene(20%)

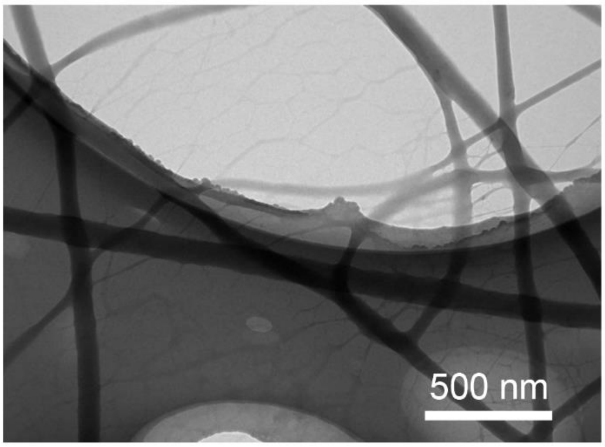

Fig. S8 TEM image of WGID membrane with 500 nm scale bar

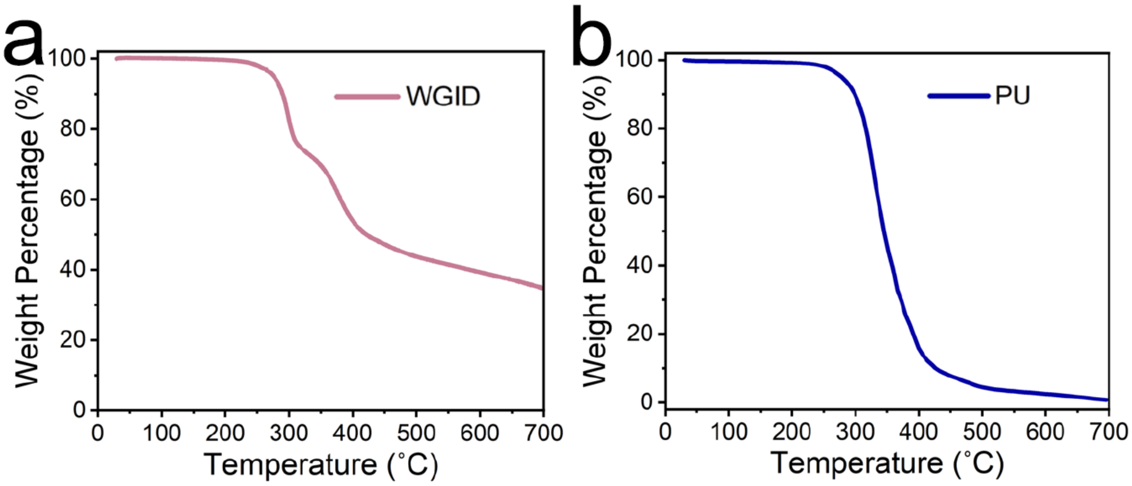

Fig. S9 TGA curves of **a** the WGID membrane, **b** PU membrane

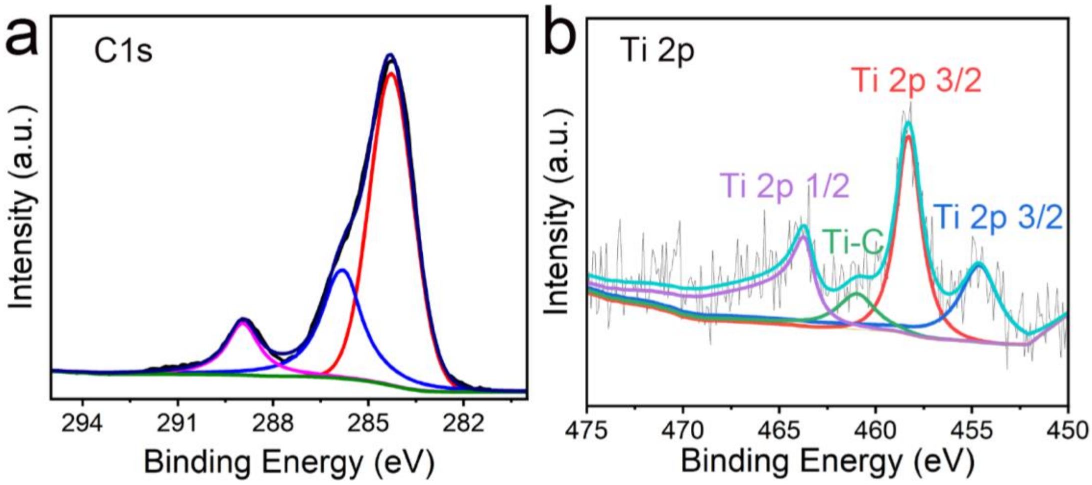

Fig. S10 XPS survey spectra of WGID membrane. **a** C 1s, and **b** Ti 2p high-resolution spectra

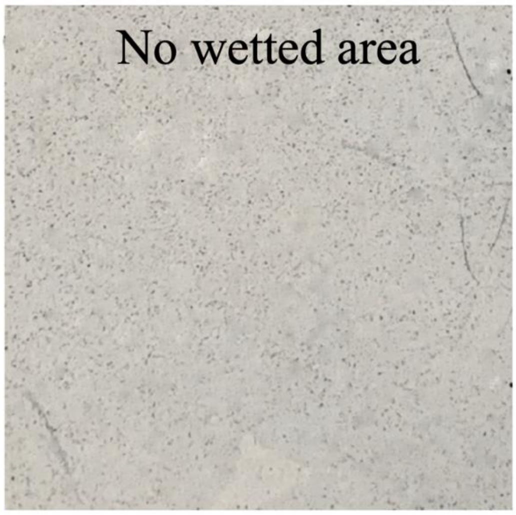

Fig. S11 Backside Photograph of WGID when water droplet was landed from hydrophilic side

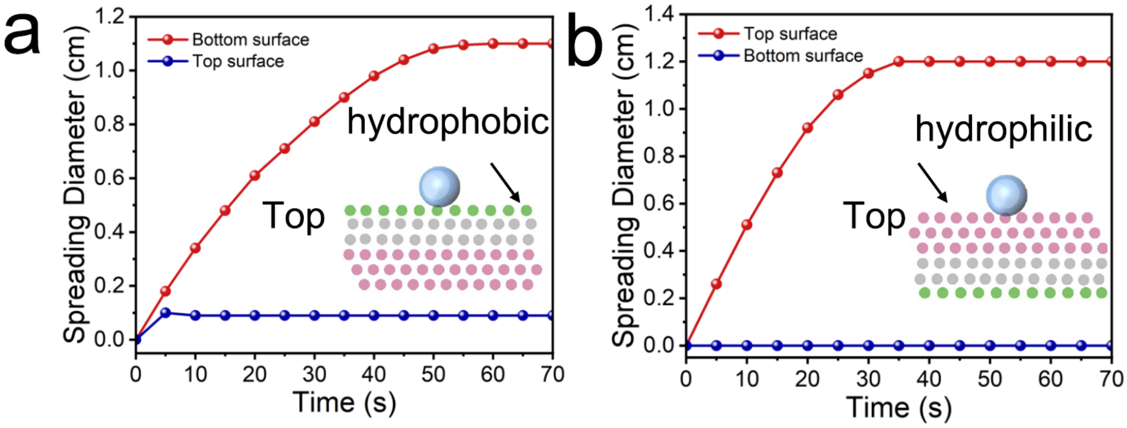

Fig. S12 Spreading diameter of the WGID membrane from different sides. **a** from hydrophobic side, **b** from hydrophilic side

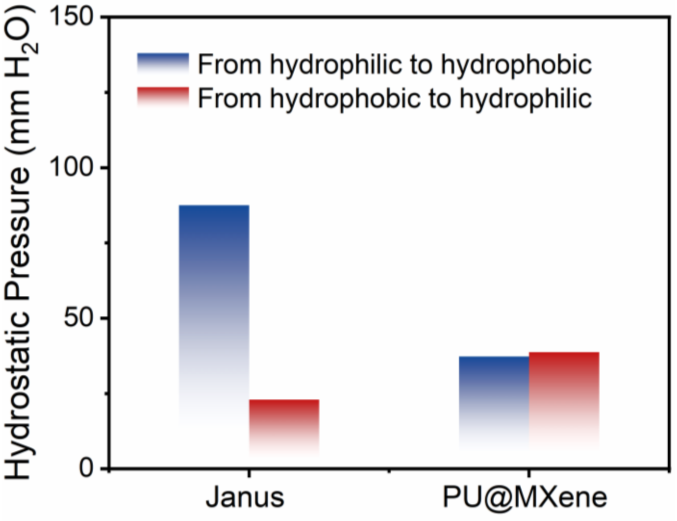

Fig. S13 Hydrostatic pressure of Janus membrane and PU@MXene(10%) membrane from both sides

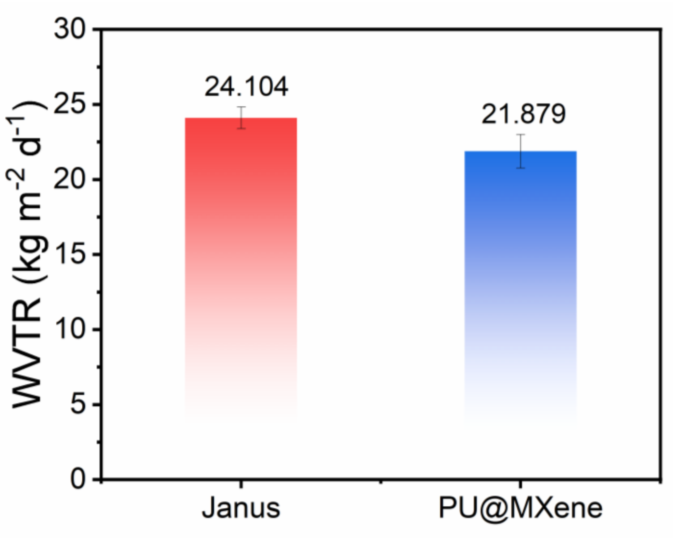

Fig. S14 WVT of Janus membrane and PU@MXene(10%) membrane

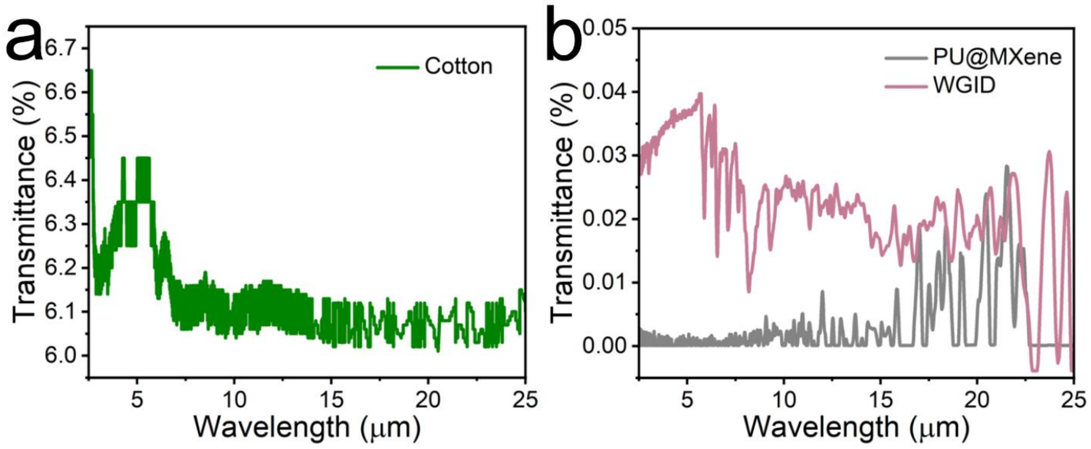

Fig. S15 The transmittance of **a** traditional cotton, **b** PU@MXene(20%) and the WGID membrane

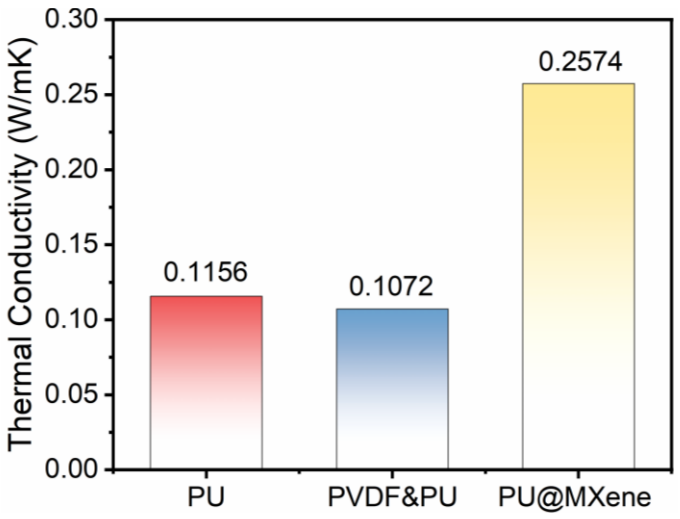

Fig. S16 Thermal conductivity of PU, PVDF&PU, and PU@MXene(10%)

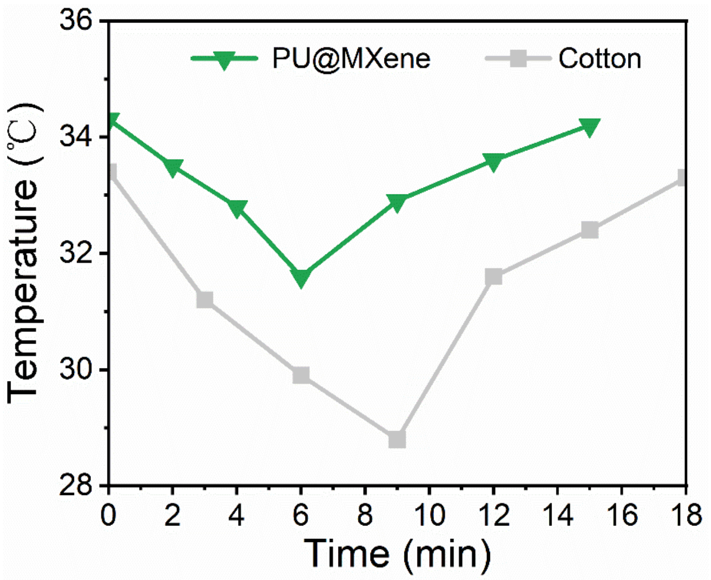

Fig. S17 Temperature change of PU@MXene, cotton.

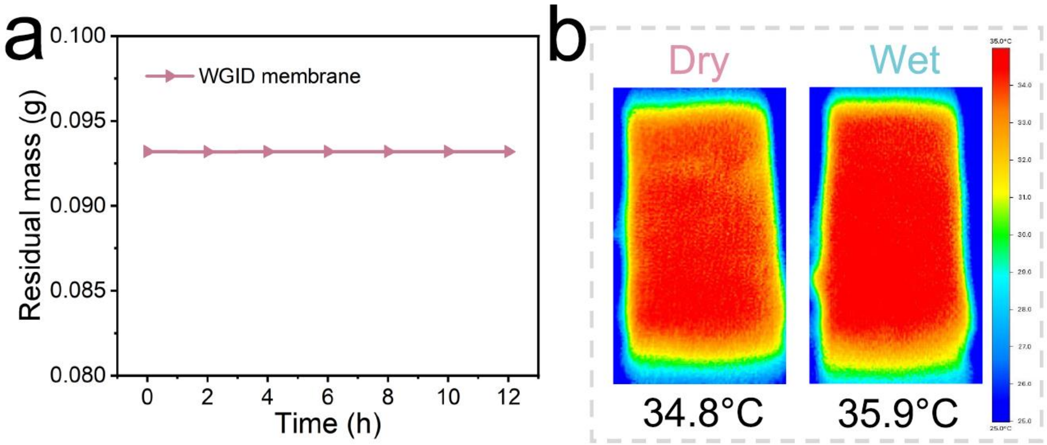

Fig. S18 a Residual mass of WGID membrane with processing different stirring time. **b** Thermal infrared images from dry state to wet state after washing for 12 hours

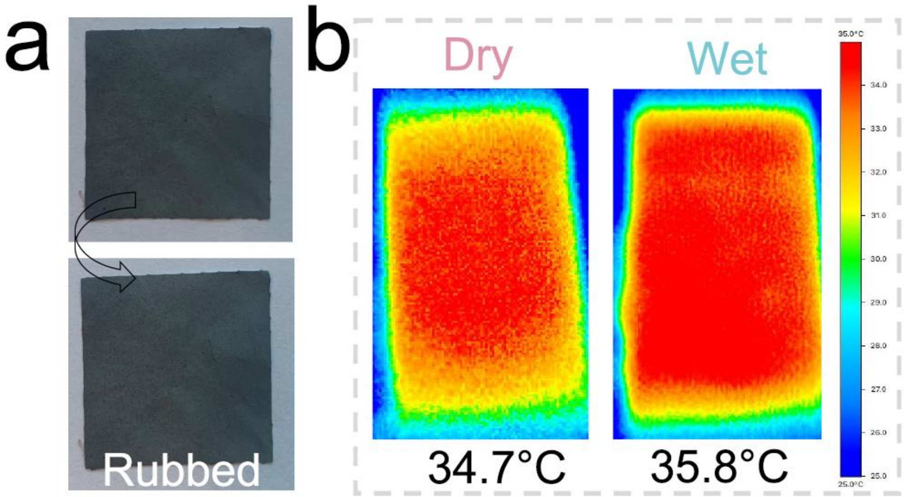

Fig. S19 a Photographs, **b** Thermal infrared images from dry state to wet state of the WGID membrane before and after being rubbed
